# A Whole Virus Pandemic Influenza H1N1 Vaccine Is Highly Immunogenic and Protective in Active Immunization and Passive Protection Mouse Models

**DOI:** 10.1371/journal.pone.0009349

**Published:** 2010-02-23

**Authors:** Otfried Kistner, Brian A. Crowe, Walter Wodal, Astrid Kerschbaum, Helga Savidis-Dacho, Nicolas Sabarth, Falko G. Falkner, Ines Mayerhofer, Wolfgang Mundt, Manfred Reiter, Leopold Grillberger, Christa Tauer, Michael Graninger, Alois Sachslehner, Michael Schwendinger, Peter Brühl, Thomas R. Kreil, Hartmut J. Ehrlich, P. Noel Barrett

**Affiliations:** Global Research and Development, Baxter BioScience, Vienna, Austria; National Institute of Allergy and Infectious Diseases, United States of America

## Abstract

The recent emergence and rapid spread of a novel swine-derived H1N1 influenza virus has resulted in the first influenza pandemic of this century. Monovalent vaccines have undergone preclinical and clinical development prior to initiation of mass immunization campaigns. We have carried out a series of immunogenicity and protection studies following active immunization of mice, which indicate that a whole virus, nonadjuvanted vaccine is immunogenic at low doses and protects against live virus challenge. The immunogenicity in this model was comparable to that of a whole virus H5N1 vaccine, which had previously been demonstrated to induce high levels of seroprotection in clinical studies. The efficacy of the H1N1 pandemic vaccine in protecting against live virus challenge was also seen to be equivalent to that of the H5N1 vaccine. The protective efficacy of the H1N1 vaccine was also confirmed using a severe combined immunodeficient (SCID) mouse model. It was demonstrated that mouse and guinea pig immune sera elicited following active H1N1 vaccination resulted in 100% protection of SCID mice following passive transfer of immune sera and lethal challenge. The immune responses to a whole virus pandemic H1N1 and a split seasonal H1N1 vaccine were also compared in this study. It was demonstrated that the whole virus vaccine induced a balanced Th-1 and Th-2 response in mice, whereas the split vaccine induced mainly a Th-2 response and only minimal levels of Th-1 responses. These data supported the initiation of clinical studies with the same low doses of whole virus vaccine that had previously been demonstrated to be immunogenic in clinical studies with a whole virus H5N1 vaccine.

## Introduction

In April 2009, an outbreak of influenza in North America was found to be caused by a new strain of influenza virus [1]. This swine-origin influenza virus was determined to be a novel strain of A/Influenza H1N1 serotype which had been derived by reassortment of swine, avian and human influenza viruses. The virus rapidly spread to a large number of countries and on June 11, 2009 the WHO declared that the infections caused by the new strain had reached pandemic proportions. As of end of January, 2010, WHO has reported approx. 14700 deaths in more than 209 countries resulting from pandemic influenza H1N1 [2]. However, given that countries are no longer required to test and report individual cases, the number of reported cases significantly understates the real number of cases.

Most confirmed cases of pandemic H1N1 infection have been characterized by self-limited, uncomplicated febrile respiratory illness and symptoms similar to those of seasonal influenza. However a substantial number of hospitalized individuals did not have underlying health issues, suggesting that the pathogenic potential of this pandemic H1N1 virus may be different to that of seasonal influenza virus strains [3].

Immunization provides the best preventive strategy against influenza virus illness. The current trivalent vaccine is unlikely to provide significant protection against the novel pandemic H1N1 strain. It has been reported that previous vaccination of children with trivalent vaccine of the last four seasons i.e. 2005–2006 to 2008–2009, did not elicit a cross-reactive antibody response to the pandemic H1N1 strain [4]. Thus a monovalent vaccine based on the novel H1N1 strain will be required to induce protective immunity. Standard H1N1 vaccine components of the trivalent seasonal vaccine consist of 15 µg hemagglutinin (HA) of split or subunit non-adjuvanted preparations. However, it has been reported that up to 90 µg of a non-adjuvanted subvirion candidate pandemic H5N1 vaccine was required to induce putative protective immune response levels in 58% of subjects after two immunizations [5]. It has however been reported that 7.5 µg HA of a whole virus H5N1 vaccine induced seroneutralizing responses in 76% of subjects after two immunizations [6] and it has also been reported that whole virus trivalent seasonal influenza vaccines are more immunogenic than non-adjuvanted subvirion vaccines [7]. Based on these reports, a whole virus H1N1 vaccine was developed with the expectation that this would facilitate substantial antigen sparing, which would allow the availability of larger amounts of vaccine compared to the use of subvirion technology. The first pre-clinical immunogenicity and animal protection studies described here suggest that this H1N1 vaccine will be effective in protecting against pandemic influenza illness in humans.

## Materials and Methods

### Ethics Statement

All animal experiments were reviewed by the Institutional Animal Care and Use Committee and approved by the Austrian regulatory authorities. All animal experiments were conducted in accordance with Austrian laws on animal experimentation and guidelines set out by the Association for Assessment and Accreditation of Laboratory Animal Care International (AAALAC) and the Office of Laboratory Animal Welfare (OLAW). Animals were housed according to OLAW and AAALAC guidelines, in housing facilities accredited by the AAALAC.

### Vaccine Strains and Reagents

Influenza viruses (A/California/07/2009 (H1N1; CDC# 2009712112), A/Vietnam/1203/2004 (H5N1; CDC#2004706280) and A/Brisbane/59/2007 (H1N1; NIBSC 07/346)), hemagglutinin (HA) antigens (A/California/7/2009 (NIBSC 09/146), A/Brisbane/59/2007 (NIBSC 08/100) and A/Vietnam/1203/2004 (CBER #50)), and antisera (A/California/7/2009 (NIBSC 09/152), A/Brisbane/59/2007 (NIBSC 08/112) and A/Vietnam/1203/2004 (CBER #S-APS1 L2)) were obtained from the Centers for Disease Control and Prevention (CDC, Atlanta, USA), the National Institute for Biological Standards and Control (NIBSC, UK), or the Center for Biologics Evaluation and Research (CBER). Baculovirus-derived recombinant HA (rHA) antigens were purchased from Protein Sciences (UK).

### Vaccine Production

Viruses amplified in serum protein free Vero cells in 100 L or 6000 L bioreactors were harvested, double-inactivated and purified as previously described. This involved use of a double inactivation procedure as established previously for H5N1 vaccines in order to ensure an extremely high margin of safety for this highly pathogenic virus [8]–[10]. Hemagglutinin (HA) antigen content was determined by single radial immunodiffusion assay [11] for A/Brisbane/59/2007 and A/Vietnam/1203/2004 and by HPLC analysis for A/California/07/2009 vaccines.

### Immunization and Challenge of CD1 Mice

Female CD1 mice (6–9 weeks old) were subcutaneously (s.c.) injected with vaccine or buffer on days 0 and 21. Functional HA-specific antibody titers were determined via hemagglutination inhibition (HI) or micro neutralization (MN) assays on days 21 and 42. On day 42, mice were challenged intranasally with 1×10^5^ TCID_50_ units of wild-type virus. Mice administered H5N1 virus were monitored for 14 days post-challenge for disease symptoms and death. Mice administered H1N1 virus were euthanized 3 days post-challenge and infectious virus in lung tissue was detected using a TCID_50_ assay. The protective dose 50% (PD_50_) was determined by survival or undetectable virus titers in the lungs following challenge with H5N1 or H1N1 virus, respectively. In addition, 100 µl of the lung tissue samples were applied directly to Roux flasks (75 cm^2^, NUNC, Cat.-No 178905) followed by a complete medium change 1 hour after inoculation. The cells were screened for CPE after 6 days of incubation at 37°C to overcome the cytotoxic effects seen at higher concentrations in the TCID_50_ assay.

### Immunizations of Balb/c Mice for Analysis of Cellular and Humoral Immunity

Balb/c mice (8–10 weeks old) were injected s.c. either once (on day 0) or twice (on days 0 and 21) with 3.75 µg of vaccine or buffer. On day 42, eye bleeds were taken by orbital puncture for IgG subclass and HI titer determinations. On days 7 and 42, spleens were obtained from euthanized animals for IFN-γ and IL-4 ELISPOT analyses.

### Microneutralization (MN) Assay

Functional H5N1 HA-specific antibody titers were determined using an MN assay, as previously described [10], and the effective dose fifty percent (ED_50_) required to induce neutralizing antibody titers of ≥20 was determined.

### Hemagglutination Inhibition (HI) Assay

Functional H1N1 HA-specific antibody titers were determined by HI assay using chicken erythrocytes, and the ED_50_ required to induce seroprotection was determined. Sera giving a negative signal in the first dilution (<1∶10) were assigned a nominal HI score of 1∶5. HI titers are expressed as reciprocal of serum dilution. Animals with a serum HI titer of ≥40 were considered seroprotected.

### TCID_50_ Assay (Tissue Culture Infectious Dose 50%)

Serial ten-fold dilutions of virus-containing samples were inoculated into 96-well microtiter plates seeded with Madin-Darby canine kidney (MDCK) cells, and incubated for 5–6 days at 37°C. Cytopathic effects in individual wells were determined via light microscopy.

### IFN-γ and IL-4 ELISPOT Assay

The frequency of IFN-γ- or Interleukin-4 (IL-4)-secreting cells was analyzed using mouse IFN-γ and IL-4 enzyme-linked immunospot (ELISPOT) kits (Mabtech AB, Nacka, Sweden), as previously described [10], using vaccine antigen at a concentration of 0.3 µg HA/ml.

### IgG Subclass Determination

ELISA plates were coated overnight at 4°C with either rHA or polyclonal anti-mouse Fab2 IgG (Sigma). After blocking non-specific binding and subsequent washing, diluted sera or serial dilutions of purified murine IgG1, IgG2a or IgG2b (Sigma) were added to the wells containing rHA or Fab2 IgG, respectively. Plates were incubated for 1 h at room temperature, washed again, prior to further incubation for 1 h with IgG subclass-specific peroxidase-conjugated goat anti-mouse IgG antibodies. Bound IgG subclass antibodies were detected colorimetrically using TMB substrate.

### H1N1 Challenge and Passive Protection of SCID Mice

Female severe combined immunodeficient (SCID) 4–5 week old mice (strain CB17/Icr-Prkdc^scid^/IcrCrl) were intranasally challenged with 1×10^5^ TCID_50_ units of wild-type virus, and monitored for 30 days for disease symptoms and death. For passive immunization, 200 µl of either naive mouse, or immunized mouse or guinea pig sera were injected intraperitoneally at days 0 and 1 prior to intranasal challenge at day 2. Immune sera were generated in CD1 mice or guinea pigs immunized twice with 3.75 µg H1N1 vaccine at days 0 and 21, and serum pools were obtained at day 42.

### Statistical Analyses

PD_50_, ED_50_ and TCID_50_ values were calculated using in-house software based on the one-hit model [12].

## Results

### 1. Dose-Dependent Immunogenicity of H1N1 Vaccine in Mice

To provide initial guidance with respect to dosing for clinical trials, groups of ten mice were immunized twice, with a three week interval and with five-fold dilutions of antigen doses ranging from 3.75 µg to 0.0012 µg HA. Hemagglutination inhibition (HI) titers were determined in the individual mice and a geometric mean titer (GMT) was determined for each dosage group, 21 days after the first and second immunization (Table 1). Seroconversion, as defined by an HI geometric mean titer ≥40, was achieved after a single dose with the 3.75 µg, 0.75 µg and 0.15 µg formulations (GMT's of 139, 86 and 53 respectively). These titers had increased substantially when measured 21 days after booster immunization. The highest mean GMT (970) was achieved 21 days after booster immunization with the 3.75 µg formulation. All individual mice demonstrated an HI titer ≥40 with as little as 0.75 µg HA antigen after one immunization and 0.03 µg after two immunizations.

**Table 1:** Dose-dependent immunogenicity of H1N1 A/California/7/2009 candidate vaccine in mice.

A/California/7/2009 Dose/µg HA	d21		d42	
	%SC	GMT	%SC	GMT
3,75 µg	90%	139	100%	970
0,75 µg	100%	86	100%	537
0,15 µg	70%	53	100%	343
0,03 µg	40%	15	100%	243
0,006 µg	10%	7	50%	32
0,0012 µg	0%	5	0%	5
Buffer	0%	5	0%	5
ED_50_	57 ng		7 ng	

CD1 mice were immunized twice with different doses of the candidate vaccine, and HI titers were determined 21 days after the first (d21) and 21 days after the booster immunization (d42) to calculate the percentage of seroconversion (%SC), geometric mean titers (GMT), and effective dose 50 (ED_50_) based on an HI titer of ≥40.

### 2. Dose Dependent Protection of Mice by H1N1 Candidate Vaccine

To evaluate the ability of the H1N1 vaccine to induce protective immune responses, a mouse challenge and protection model was established by intra-nasal challenge of CD1 and Balb/c mice with virus titers ranging from 10^2^ to 10^5^ TCID_50_. No lethality was observed at any challenge dose although clear symptoms of ruffled hair and buckled back could be observed within two days after infection. This was in agreement with a previous report for infection of Balb/c mice with the novel H1N1 virus [13]. Maximum virus titers could be determined in the lungs of infected animals three days after challenge with 10^5^ TCID_50_ of H1N1 virus in the CD-1 mice (data not shown). This model was then utilized to investigate the protective efficacy of the candidate vaccine. The out-bred CD-1 strain was also considered to better reflect the human genetic situation than the inbred Balb/c mouse strain. Groups of 5 mice were s.c. immunized twice with decreasing doses of vaccine ranging from 3.75 µg to 0.0012 µg antigen before being challenged intra-nasally with 10^5^ TCID_50_ of H1N1 A/California/7/2009, 21 days after the booster immunization. Three days after challenge, the lungs of immunized and control mice were harvested and virus titers were determined (see Methods. Maximum virus titers of 10^5.5^ TCID_50_/ml of resuspended lung tissue were obtained for control animals after challenge while a titer less than <10^1.1^ TCID_50_/ml (level of detection due to toxicity of lung tissue in tissue culture) was determined to be indicative of protection.

**Figure 1:**
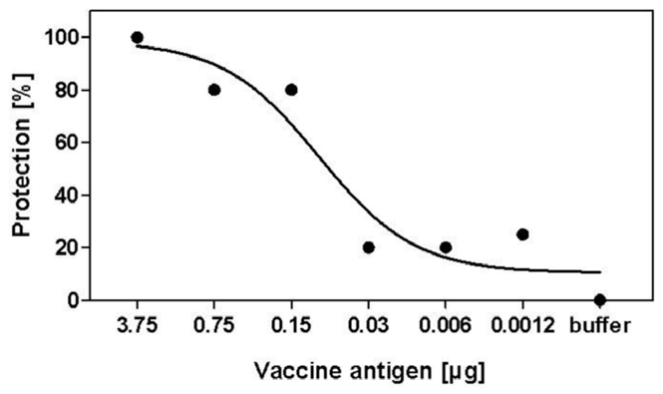
Protection of mice from lung viremia. Groups of CD1 mice were immunized twice with five-fold serial dilutions of pandemic H1N1 (H1N1 A/California/7/2009) whole virus vaccine, before being challenged intranasally with 10^5^ TCID_50_. Lungs were harvested at day three after challenge, and virus titers determined as described (Methods). Lack of detection of virus in lungs was considered indicative of protection.

The data presented in Fig. 1 demonstrate that 100% protection with respect to undetectable virus titers in lung tissue could be obtained following immunization with two doses of 3.75 µg HA antigen whereas 80% protection could be obtained with as little as 0.15 µg.

### 3. Effective Dose 50% (ED_50_) and Protective Dose 50% (PD_50_) Comparison between Seasonal and Pandemic H1N1 and H5n1 Vaccines

As no data is available about the efficacy of the present generation of candidate pandemic vaccines, comparative immunogenicity studies were done with an H5N1 vaccine and an H1N1 component (A/Brisbane/59/2007) of a standard seasonal vaccine, both of which have been in extensive clinical trials and have been demonstrated to be highly immunogenic with high levels of seroconversion and seroprotection in humans [6], [14], [15]. ED_50_ and PD_50_ determinations were calculated for the three vaccines after immunization of groups of mice with two doses of serial dilutions of vaccine antigen as described in Methods. These data (Table 2) demonstrate that the three vaccines were comparable with respect to immunogenicity with mean ED_50_ values of 15 ng, 13 ng and 34 ng being obtained for H1N1 Brisbane, H1N1 California and H5N1 Vietnam. PD_50_ values could not be determined for the seasonal H1N1 Brisbane strain because of the lack of an appropriate challenge model but comparison of the two pandemic strain vaccines demonstrated similar levels of protection with values of 5 ng and 8 ng being obtained for the H1N1 and H5N1 vaccines respectively.

**Table 2:** ED_50_ and PD_50_ comparison between seasonal and pandemic H1N1, and pandemic H5N1 vaccines.

Vaccine	ED_50_			PD_50_		
	mean	SD	N	mean	SD	N
H1N1 A/Brisbane/59/2007	15 ng^1^	4	2	n.a.	n.a.	n.a.
H1N1 A/California/7/2009	13 ng^1^	11.4	3	5 ng^3^	3.6	3
H5N1 A/Vietnam/1203/2004	34 ng^2^	21	6	8 ng^4^	6	9

n.a. not applicable (no challenge model available).

1 based on an HI titer of ≥40.

2 based on a MN titer of ≥20.

3 based on the titer of infectious virus in the lungs of mice 3 days after challenge.

4 based on survival of mice 14 days after challenge.

Groups of mice were immunized twice with serial dilutions of antigen doses up to 5 µg of seasonal H1N1 (H1N1 A/Brisbane/59/2007), pandemic H1N1 (H1N1 A/California/7/2009) and pandemic H5N1 (H5N1 A/Vietnam/1203/2004) vaccines. The minimum antigen dose that resulted in seroconversion (ED_50_) or protection against challenge (PD_50_) of 50% of immunized animals was determined, and is given as mean with standard deviation (SD) and number of study replicates tested (N).

### 4. T helper Cell and IgG Subclass Responses in Mice

**Figure 2:**
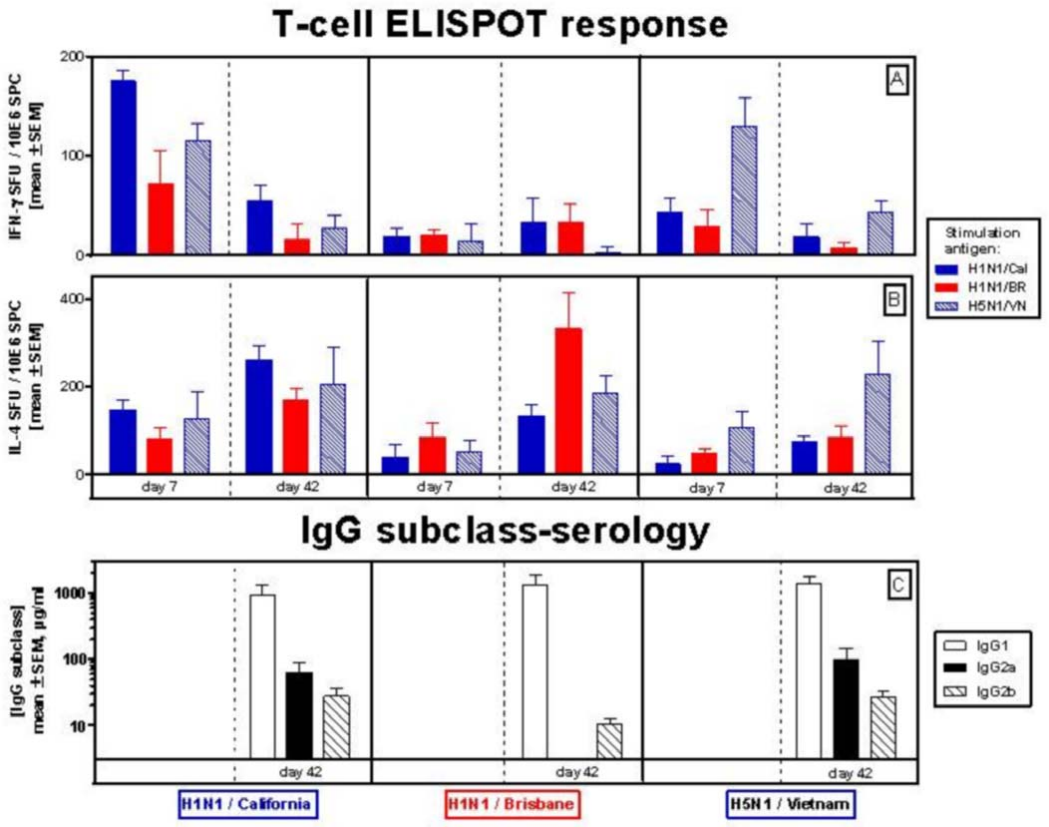
Th-1 and Th-2 cytokine responses in mice immunized with seasonal and pandemic H1N1, and pandemic H5N1 vaccines. Balb/c mice were immunized with pandemic H1N1 (H1N1 A/California/7/2009), seasonal H1N1 (H1N1 A/Brisbane/59/2007), and pandemic H5N1 (H5N1 A/Vietnam/1203/2004) vaccines. Spleen cells were collected 7 days after the first, or 21 days after the booster immunization (i.e. 42 days after the first), and stimulated with various seasonal or pandemic influenza virus antigens, before determination of cells responding by secretion of either IFN-g or IL-4 by an ELISPOT assay. Anti-HA IgG subclass responses were analyzed by ELISA using sera collected on day 42.

The type of cellular immune response induced by the pandemic H1N1/California vaccine was characterized and compared with that produced by the pandemic H5N1/Vietnam and seasonal H1N1/Brisbane vaccines using inbred Balb/c mice. After each immunization, homologous and heterologous T-helper cell responses were evaluated by IFN-g and IL-4 ELISPOT analyses as described in Methods. Spleen cells were collected 7 days after the first and 21 days after the second immunization (day 42) and stimulated with seasonal or pandemic influenza virus antigens. The Th-1 (IFN-g) responses were found to be highest on day 7 (Fig. 2A), while Th-2 (IL-4) responses were higher on day 42 (Fig. 2B). In the case of the H1N1 California immunized mice, the highest Th-1 type responses were obtained following stimulation with the homologous antigen, although substantial cross-reactive responses were also seen after stimulation with H5N1 Vietnam and to a lesser extent following H1N1 Brisbane stimulation. Numbers of IFN-g secreting cells specific for the homologous antigen were comparable in mice immunized with pandemic whole viral vaccines, but lower for the mice immunized with the split influenza vaccine.

The Th-2 responses to H1N1 California showed a similar picture with the highest IL-4 response directed against the homologous antigen. Substantial cross-reactive responses were also seen after stimulation with the whole virus H5N1 Vietnam and split H1N1 Brisbane. Again, the homologous Th-2 responses of the two whole virus pandemic vaccines were comparable, while that to seasonal H1N1 Brisbane, in this case, was slightly higher. Overall, the whole viral vaccines induced similar numbers of IFN-g and IL-4 secreting cells on day 7, indicating an initial mixed Th-1/Th-2 response, which shifted after the booster immunization to a predominant Th-2 response. In contrast, there was little or no Th-1 response induced by the split H1N1 Brisbane vaccine on Day 7, and thus a Th-2 response bias was observed already on day 7, after the first immunization.

To further substantiate the difference between the type of cellular responses elicited by the whole viral pandemic H1N1 California and H5N1 Vietnam vaccines in comparison to the split seasonal H1N1 Brisbane vaccine, anti-HA IgG subclass responses were analyzed by ELISA as described in Methods, using sera collected on day 42. While anti-HA IgG1 subclass antibody responses, characteristic of a Th-2 type response, were dominant for all 3 vaccines (Fig. 2C), substantial IgG2a and IgG2b subclass antibody responses, characteristic of Th-1 responses, were only detected in mice immunized with the whole viral pandemic vaccines. This indicates that whole virus vaccines are capable of inducing both Th-1 and Th-2 responses, whereas the split vaccine produces strong Th-2 responses with only minimum levels of Th-1 type T cell or Th-1 driven IgG subclass responses.

### 5. Protection of SCID Mice by Passive Transfer of Immune Serum

The results of the immunization and challenge studies reported in Fig. 1 demonstrated that the H1N1 vaccine was capable of preventing virus replication in the lungs after challenge with high titer H1N1 virus. These studies were further extended to demonstrate the protective efficacy of the vaccine using a SCID mouse model which was more sensitive to H1N1 infection. Intra-nasal (i.n.) challenge of SCID mice with H1N1 resulted in infection with a dose dependent lethality, unlike the situation with Balb/c or CD 1 mice where no lethality was observed. Lethal infection of SCID mice was achieved after i.n. infection with a LD_50_ of 3.8±0.3 log_10_ TCID_50_ (mean ± SD, N = 2). After infection with 10^5^ TCID_50_ all mice died, with a mean survival time of 18 days. This sensitive model was therefore utilized to determine the protective efficacy of vaccination using a passive transfer model. Following immunization of mice and guinea pigs with the H1N1 vaccine, sera pools were prepared and H1N1 specific HI titers were determined. An HI titer of 640 was measured in both mouse and guinea pig preparations. Groups of 6 SCID mice were twice injected i.p. with 200 µl of immune sera from both pools or with naïve mouse serum, 24 hours apart. One day after the second passive transfer these groups and a control non-treated group of mice were i.n. challenged with 10^5^ TCID_50_ H1N1 in a 20 µl volume.

**Figure 3:**
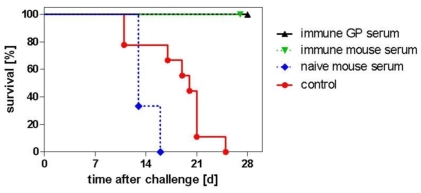
H1N1 challenge and passive protection of SCID mice. SCID mice were challenged with 10^5^ TCID_50_ pandemic H1N1 (H1N1 A/California/7/2009) by intranasal instillation, and survival monitored for 30 days. For passive protection, 200 µl immune mouse or guinea pig (GP) sera, or naïve mouse serum, were intraperitoneally administered to mice both at days one and two prior to virus challenge.

The data presented in Fig. 3 demonstrates that passive transfer of serum from either mice or guinea-pigs immunized with the A/California/07/2009 H1N1 vaccine resulted in complete protection with no lethalities over a 30 day observation period. In contrast all mice which received naïve serum or were in a control non-treated group died between 13 and 25 days. Clinical monitoring for disease symptoms (ruffled fur, hunched back, exhaustion) was also carried out over a period of 30 days. While the control mice demonstrated symptoms by day 10 following challenge, no clinical symptoms were observed in the mice receiving anti-H1N1 sera.

At the end of the experiment at day 30, lungs of the surviving animals were collected. All these animals were symptom-free at this stage but all had virus titers detectable in the lungs. Mean titers of 10^4.2^ and 10^4.0^ TCID_50_ were measured in mice treated with mouse or guinea-pig antisera respectively, whereas in non-treated controls, examined at first onset of symptoms, the titers were 2 to 3 logs higher.

## Discussion

This study was designed to assess the immunogenicity and protective efficacy of a candidate vaccine against the pandemic A/California/07/2009 pandemic H1N1 virus in animal models, prior to initiation of human clinical studies. It had been previously reported that a candidate pandemic H5N1 vaccine produced by an identical process was highly immunogenic and protective in mouse models [9]. These studies were subsequently demonstrated to be highly predictive of the immunogenicity demonstrated in human trials, particularly with respect to immunogenicity at low doses and the lack of immune enhancement by use of an alum based adjuvant [7].

As such it was considered that similar studies would be valuable in assessing the optimal dosing for clinical trials with the H1N1 candidate vaccine. In addition although surrogate serological markers for seasonal influenza vaccine have been established [16], no clear cut correlate for protection has been established for potential pandemic vaccines such as H5N1 or novel H1N1 strain vaccines. Thus data obtained from animal protection studies could be of value in combination with data obtained from human dose-finding and observational efficacy studies following vaccine use in a pandemic situation such as presently exists for the novel H1N1 virus.

The data illustrated in Table 1 was encouraging in that immunization with as little as 30 ng HA antigen resulted in a mean GMT of 243 as measured in the HI assay after two immunizations. Following a single dose immunization regimen as little as 0.15 µg still resulted in a mean HI titer response above the accepted 1∶40 threshold for seroprotection in humans for seasonal influenza vaccines. These data with a non-adjuvanted whole virus H1N1 vaccine were similar or superior to those obtained for a MF59 adjuvanted subunit H1N1 vaccine in mouse studies, where 0.5 µg induced an average HI titer of 63 after one immunization. In contrast the non-adjuvanted split vaccine used in those studies resulted in substantially lower HI titer responses [17]. The immunogenicity of the MF59 adjuvanted vaccine has been subsequently confirmed in human studies with a 7.5 µg dosage resulting in 80% seroprotection after a single immunization [18].

The immunogenicity described in Table 1 was also supported by the data obtained in challenge studies, with no virus being detected in lung tissue of 100% of mice following immunization with two doses of 3.75 µg HA antigen and 80% protection being obtained with as little as 0.15 µg.

The probability of pandemic H1N1 vaccine efficacy in humans was further analyzed by comparison with the immunogenicity of a seasonal monovalent H1N1 vaccine (A/Brisbane/59/2007) which was demonstrated to be highly immunogenic as a component of a trivalent split seasonal vaccine in multiple human trials [14] and with a whole virus H5N1 vaccine which was also highly immunogenic in human trials [6], [15] and highly protective in pre-clinical lethal mouse challenge studies [10]. The data presented in Table 2 demonstrated that the three vaccines were comparable with respect to immunogenicity with mean ED_50_ values of 15 ng, 13 ng and 34 ng being obtained for the seasonal H1N1 Brisbane, the H1N1 California and the H5N1 Vietnam vaccines respectively. The H1N1 California and H5N1 vaccines were also demonstrated to be highly comparable with respect to protective efficacy with PD_50_ values of 5 ng and 8 ng being obtained for the H1N1 and H5N1 vaccines respectively (Table 2).

The ability of the H1N1 vaccine to prevent virus replication in the lungs of mice may be a predictive marker for disease prevention in humans. However, the inability of the virus to induce lethality in the immune competent mouse model prevented a clear cut assessment of efficacy. Other standard influenza animal models such as the ferret also do not consistently display clear clinical signs of infection, and is also not a lethal model for H1N1 California [13], [19], [20]. In contrast, the SCID mouse was susceptible to lethal infection but because of its intrinsic immune deficient nature could not be utilized as a model for active immunization studies. This model was therefore adapted to indirectly measure the efficacy of the vaccine in passive transfer studies. The data presented in Fig. 3 confirm the protective potential of this vaccine in that immune sera generated by active immunization of mice and guinea-pigs resulted in 100% protection of susceptible SCID mice following passive transfer whereas 100% of control mice succumbed to lethal infection following challenge with 10^5^ TCID_50_ of infectious virus.

In this study monitoring was discontinued at day 30 i.e. 5 days after the last death in the control group. All surviving animals were symptom-free at this stage but following sacrifice it could be demonstrated that a significant virus titer was present in the lungs, although the virus load was substantially reduced compared to that detected in control, non-treated animals. This data confirms reports that it is difficult to obtain full virus clearance from the lungs of SCID mice. It has been reported that only high concentrations of anti-HA specific cocktails of mouse monoclonal antibodies transferred repeatedly resulted in full virus clearance in immune deficient mice in an influenza treatment model [21], [22]. As such it is possible that disease and death may have occurred in the healthy SCID mice at a later stage following decline in titers of passively transferred antibody. However, a recent study with Ebola virus showed that passive transfer of specific antibodies protects immune-deficient mice against lethal Ebola virus infection without complete inhibition of viral replication [23].

The superior immunogenicity of non-adjuvanted whole virus pandemic H5N1 vaccines compared to non-adjuvanted split virus vaccines has been demonstrated in clinical studies [5], [6]. In this study we compared the Th-1 and Th-2 responses to immunization with non-adjuvanted, whole virus pandemic H1N1 and H5N1 vaccines to a split virus H1N1 seasonal vaccine in Balb/c mice. The data presented in Fig. 2 demonstrate that the whole virus pandemic H1N1 and H5N1 vaccines are capable of inducing both Th-1 and Th-2 responses in mice, whereas the seasonal split H1N1 induced only minimal levels of Th-1 T-cell or Th-1 driven IgG subclass responses. This differentiated response may at least partially explain the superior immunogenicity of whole virus pandemic influenza vaccines compared to non-adjuvanted subvirion vaccines

The data reported here indicate that the whole virus pandemic H1N1 vaccine is immunogenic at low doses and protective in both active and passive transfer challenge studies. These data supported the decision to initiate clinical studies with this whole virus non-adjuvanted vaccine at low doses i.e. 7.5 µg and 3.75 µg despite the fact that non-adjuvanted split virus H5N1 vaccines have been poorly immunogenic at dosages up to 90 µg. Preliminary data from H1N1 clinical studies indicate that both the 3.75 µg and 7.5 µg non-adjuvanted formulation induced high levels of seroprotective responses (HI titer ≥1∶40) in human studies.

In addition, multiple clinical studies have recently been published, reporting that a variety of adjuvanted and non-adjuvanted split virion vaccines can also induce high levels of seroprotective responses [18], [24]–[32]. It will be highly interesting to compare field efficacy data (when available) for all of these vaccines with serological responses in human trials and immunogenicity and protection data in animal models as reported here.
